# Canonical and non-canonical JAK/STAT transcriptional targets may be involved in distinct and overlapping cellular processes

**DOI:** 10.1186/s12864-017-4058-y

**Published:** 2017-09-11

**Authors:** Amy Tsurumi, Connie Zhao, Willis X. Li

**Affiliations:** 1 Department of Surgery, Massachusetts General Hospital, 50 Blossom St., Thier 340, Boston, MA 02114 USA; 2 Department of Microbiology and Immunology, Harvard Medical School, 77 Ave. Louis Pasteur, Boston, MA 02115 USA; 3 Shriners Hospitals for Children-Boston®, 51 Blossom St, Boston, MA 02114 USA; 4 Genomics Resource Center, The Rockefeller University, New York, NY USA; 5 Department of Medicine, University of California at San Diego, 9500 Gilman Dr, La Jolla, CA 92093 USA

**Keywords:** JAK, STAT, Non-canonical JAK/STAT signaling, *Drosophila*, Development, Signal transduction, Epigenetics, Heterochromatin

## Abstract

**Background:**

The Janus kinase-signal transducer and activator of transcription (JAK/STAT) pathway has been well-characterized as a crucial signal transduction cascade that regulates vital biological responses including development, immunity and oncogenesis. Additionally to its canonical pathway that uses the phosphorylated form of the STAT transcription factor, recently the non-canonical pathway involving heterochromatin formation by unphosphorylated STAT was recently uncovered. Considering the significant role of the JAK/STAT pathway, we used the simple *Drosophila* system in which the non-canonical pathway was initially characterized, to compare putative canonical versus non-canonical transcriptional targets across the genome. We analyzed microarray expression patterns of wildtype, *Jak* gain- and loss-of-function mutants, as well as the *Stat* loss-of-function mutant during embryogenesis, since the contribution of the canonical signal transduction pathway has been well-characterized in these contexts. Previous studies have also demonstrated that *Jak* gain-of-function and *Stat* mutants counter heterochromatin silencing to de-repress target genes by the non-canonical pathway.

**Results:**

Compared to canonical target genomic loci, non-canonical targets were significantly more associated with sites enriched with heterochromatin-related factors (*p* = 0.004). Furthermore, putative canonical and non-canonical transcriptional targets identified displayed some differences in biological pathways they regulate, as determined by Gene Ontology (GO) enrichment analyses. Canonical targets were enriched mainly with genes relevant to development and immunity, as expected, whereas the non-canonical target gene set mainly showed enrichment of genes for various metabolic responses and stress response, highlighting the possibility that some differences may exist between the two loci.

**Conclusions:**

Canonical and non-canonical JAK/STAT genes may regulate distinct and overlapping sets of genes and may perform specific overall functions in physiology. Further studies at different developmental stages, or using distinct tissues may identify additional targets and provide insight into which gene targets are unique to the canonical or non-canonical pathway.

**Electronic supplementary material:**

The online version of this article (doi:10.1186/s12864-017-4058-y) contains supplementary material, which is available to authorized users.

## Background

The Janus kinase-signal transducer and activator of transcription (JAK/STAT) signaling pathway was originally characterized in mammals as an intracellular signaling pathway regulating cytokine signaling [1–5] and was later found to be highly conserved in various organisms, including *Drosophila* [6–9]. Four JAKs and seven STAT gene products have been identified in mammals, whereas in *Drosophila*, the pathway components are comprised of simply one JAK and one STAT, making it an excellent model system to study their biological roles [8, 10]. In both mammals and *Drosophila*, the canonical signaling is initiated by the binding of an extracellular ligand to transmembrane receptors that induce dimerization and activate the JAKs associated with these receptors. Then, the activated JAKs phosphorylate tyrosine residues on the cytoplasmic tails of the receptors that serve as docking sites for cytoplasmic STAT transcription factors, which then are phosphorylated, dimerize and translocate to the nucleus to activate transcription of target genes [11–13]. It has been well-established across various species that the canonical JAK/STAT pathway performs essential roles in development, immune signaling and cancers by its direct transcriptional activation of target genes [8, 14–18].

The non-canonical JAK/STAT pathway was initially identified in *Drosophila,* in which both the STAT transcription factor and the protein inhibitor of activated STAT homologue (dPIAS) were found to be suppressors of variegation, a heterochromatin-mediated phenomenon [19–21]. Heterochromatin-mediated gene silencing mechanisms involving the main non-histone key protein, Heterochromatin Protein 1a (HP1a), otherwise known as Suppressor of varigation 205 (Su(var)205) in *Drosophila*, is highly conserved across species [19, 22, 23]. HP1a recognizes and is recruited to specific genomic loci by di- and tri-methylated histone 3 lysine 9 (H3K9), which is regulated by the Su(var)3–9 methyltransferase [22, 24, 25]. The biological role of such non-canonical JAK/STAT pathway was first meticulously characterized in a tumor model of *Drosophila*, in which unphosphorylated STAT was found to exist in the nucleus and to have the ability to stabilize heterochromatin and suppress tumor growth [12, 19, 26]. Moreover, it was found that heterochromatin formation and stabilization by unphosphorylated STAT protects genome stability [27] and prolongs lifespan [28]. The pivotal role of unphosphorylated STAT in heterochromatin maintenance and tumor suppression was also recapitulated in mammals [29]. Other groups have reported that unphosphorylated STAT proteins can translocate to and prominently exist in the nucleus in various mammalian cells at quiescence, when STAT proteins are not phosphorylated [30–35]. Several studies have demonstrated functions of STAT1, STAT3, STAT5 and STAT6 in mammals that involve mechanisms that are distinct to their well-established canonical transcriptional pathway [36–45]. Additional studies have characterized the involvement of the linker histone H1 in non-canonical JAK/STAT mechanisms, further suggesting the central role of unphosphorylated STAT in epigenetic regulation [46]. In *Drosophila,* a connection between JAK/STAT signaling and chromatin remodeling has also been suggested by the finding that the transcriptional repressor Ken that recruits a nucleosome remodeling factor (NURF), shares binding sequences to suppress STAT-mediated transcription of innate immune genes [47]. The epigenetic control of heterochromatin formation and remodeling are crucial for key nuclear processes including gene silencing, chromosomal packaging and segregation during mitosis, genome stability and cell differentiation [27, 48, 49]. Additionally to its biological role in oncogenesis, heterochromatin maintenance in *Drosophila* was found to mediate lifespan [28] and stem cell maintenance [50], as well as implicated in mammalian aging [51]. These observations further underline the crucial role of the non-canonical JAK/STAT pathway in various vital biological processes, additionally to its formerly established canonical signal transduction mechanisms.

Considering the well-established role of the canonical JAK/STAT signaling cascade and the recent characterization of non-canonical JAK/STAT that is distinct and appear to perform specific roles in physiology and pathogenesis, it is important to identify target genes and genomic loci that may be regulated by one or the other. While there are previous studies that have undertaken a systematic approach to uncover JAK/STAT pathway regulators [52, 53], there has not been a study addressing differences and similarities between canonical and non-canonical targets. We therefore took a genome-wide approach to identify target loci pertaining to each, using *Drosophila* as a simple model system, in which both the canonical and non-canonical JAK/STAT have been particularly well-characterized. We analyzed genome-wide transcriptional targets of the *Jak* gain- and loss-of-function mutants, and the *Stat* loss-of-function mutant during embryogenesis, as previous studies have addressed the role of the canonical signal transduction in these mutants and furthermore, it has also been found that *Jak* gain-of-function and *Stat* loss-of-function mutants counter heterochromatin silencing to de-repress target genes by the non-canonical pathway [19, 26]. We found non-canonical targets to correlate with increased overlaps with the distribution of HP1a, Su(var)3–9 and H3K9me3, which are hallmarks of heterochromatin formation. Moreover, our study demonstrates that while canonical targets tend to be enriched with genes relevant to development and immune response, non-canonical target genes tend to be more enriched with GO terms related to metabolism and stress response, thus possibly revealing a previously unidentified putative link between the non-canonical JAK/STAT pathway and metabolism and stress response, mediated by heterochromatin. We therefore conclude that a significant number of distinct canonical and non-canonical targets appear to exist, additionally to overlapping processes and loci. Further experimental results comparing transcriptome alterations in conjunction with chromatin immunoprecipitation from the same embryonic samples will be beneficial for confirming the conclusions from these analyses.

## Results

### Analysis of various *JAK/*S*TAT* mutant embryo transcriptome changes relative to wildtype show clustering based on genotype and embryo stage

**Fig. 1:**
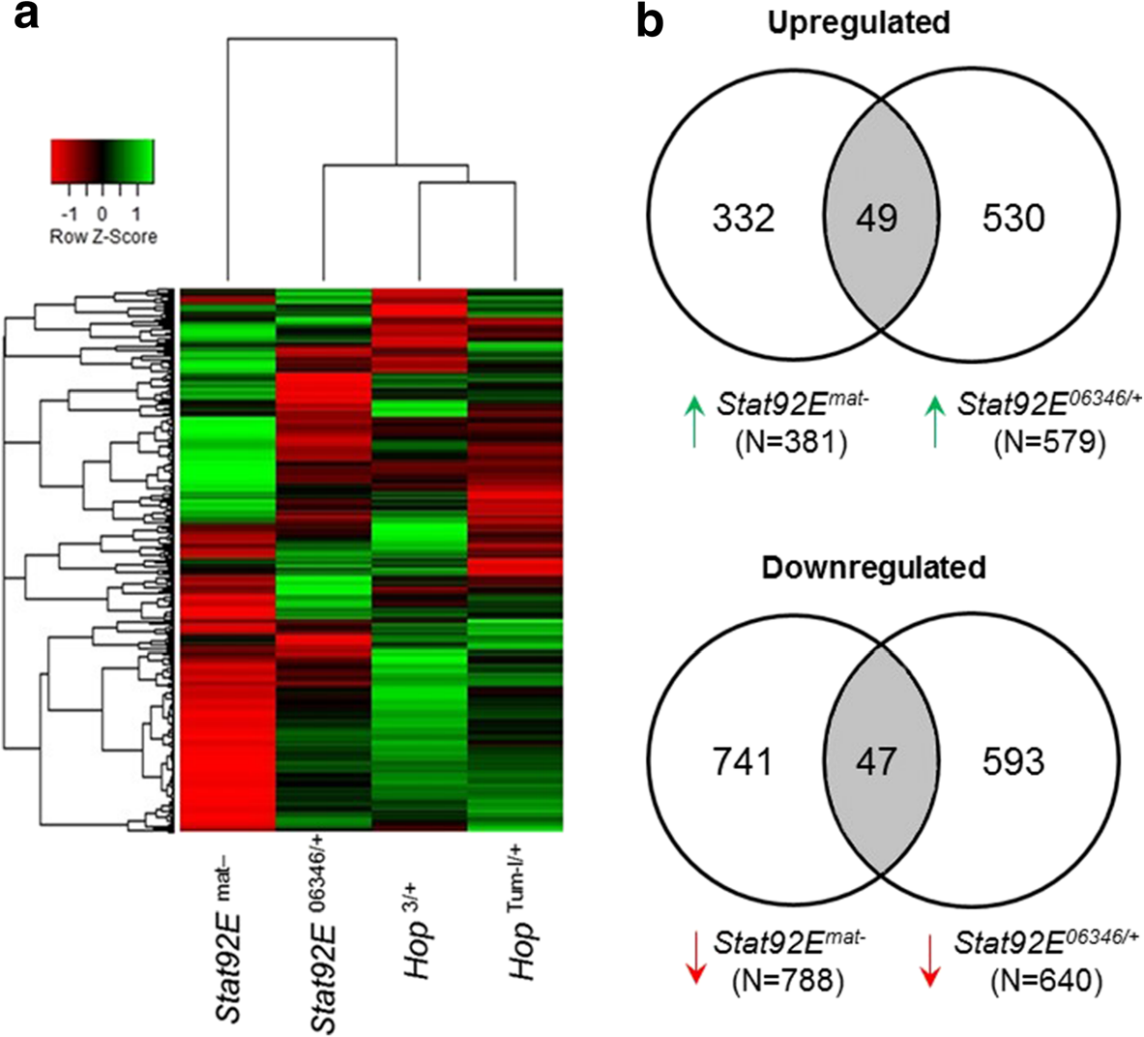
Hierarchical clustering of *Jak* gain-of-function and *Stat* loss-of-function mutants show separation by gene and stage of the embryo. (**a**) Differentially regulated genes were clustered by Pearson’s correlation complete distance separation. (**b**) Overlaps between *Stat92E* ^*mat-*^ and *Stat92E* ^*06346/+*^ embryos were determined and suggest that a large number of genes are regulated stage-specifically

We collected early *Drosophila* embryos (0–12 h eggs) from the following four different genotypes: *Jak* gain- (*hop* ^*Tum-l/+*^), and loss-of-function (*hop* ^*3/+*^), *Stat* loss-of-function heterozygotes *(Stat92E* ^*06346/+*^), and the *Stat* loss-of-function maternal null (*Stat92E* ^*mat-*^). We conducted microarray experiments to compare the transcriptome of these embryos (Additional file 1: Table S1). Gene expression levels were calculated as fold changes relative to wildtype (*w* ^*1118*^) embryos of the same stage. Hierarchical clustering analyses indicated that *hop* ^*Tum-l/+*^ and *Stat92E* ^*mat-*^ embryos were most dissimilar, with many genes expressed in the opposite manner (Fig. 1A), consistent with gain- and loss-of-function of the *JAK/STAT* pathway. Analysis of differentially regulated probe sets found clustering of *hop* ^*Tum-l/+*^ and *hop* ^*3/+*^ together, then *Stat92E* ^*06346/+*^, and *Stat92E* ^*mat-*^ (Fig. 1A; Additional file 2: Table S2). Next, we assessed the number of overlapping probe sets between *Stat92E* ^*mat-*^ and *Stat92E* ^*06346/+*^ upregulated and downregulated sets of genes (Additional file 3: Table S3). For the upregulated probe sets there were 49 that were shared between the 381 *Stat92E* ^*mat-*^ and 579 *Stat92E* ^*06346/+*^ and for the downregulated set, there were 47 that were shared between the 788 *Stat92E* ^*mat-*^ and 640 *Stat92E* ^*06346/+*^ significantly downregulated probe sets (Fig. 1B). Since *Stat92E* ^*06346/+*^ heterozygous embryos are expected to undergo normal development in spite of reduced *Stat* gene activity, the difference between the transcriptomes of *Stat92E* ^*mat-*^ and *Stat92E* ^*06346/+*^ might be due to a failure in activation of the zygotic genome in *Stat92E* ^*mat-*^ embryos, as we have previously shown [54]. The differentially regulated genes include many of the known canonical JAK/STAT pathway targets genes, such as the Turandot (Tot) family of humoral factors [55] (Additional file 2: Table S2). We validated the differential expression of several of these known JAK/STAT target genes using RT-PCR in the *hop* ^*Tum-l/+*^ overexpression mutants compared to wildtype (Additional file 4: Fig. S1). These results suggest that the activation status and levels of JAK and STAT affect the transcriptome of the early *Drosophila* embryo. In our previously published publication, we have validated reduced expression in the early loss-of-function embryo of the *dpp, Kr, tll,* and *eve* target genes that we have also found in our current microarray analysis [54].

### Canonical versus non-canonical JAK/STAT targets can be inferred from differential transcriptional analyses across the genome of various mutants

A hallmark of the noncanonical STAT pathway is that loss of STAT and JAK overactivation both result in reduced heterochromatin levels and previously demonstrated to be relevant in multiple heterochromatin-involved processes [26–28]. Thus, genes normally repressed by heterochromatin would be depressed as a result of reduced heterochromatin. These studies showed that in contrast, in the canonical pathway, loss of STAT and JAK overactivation have opposite effects on STAT target gene expression.

**Fig. 2:**
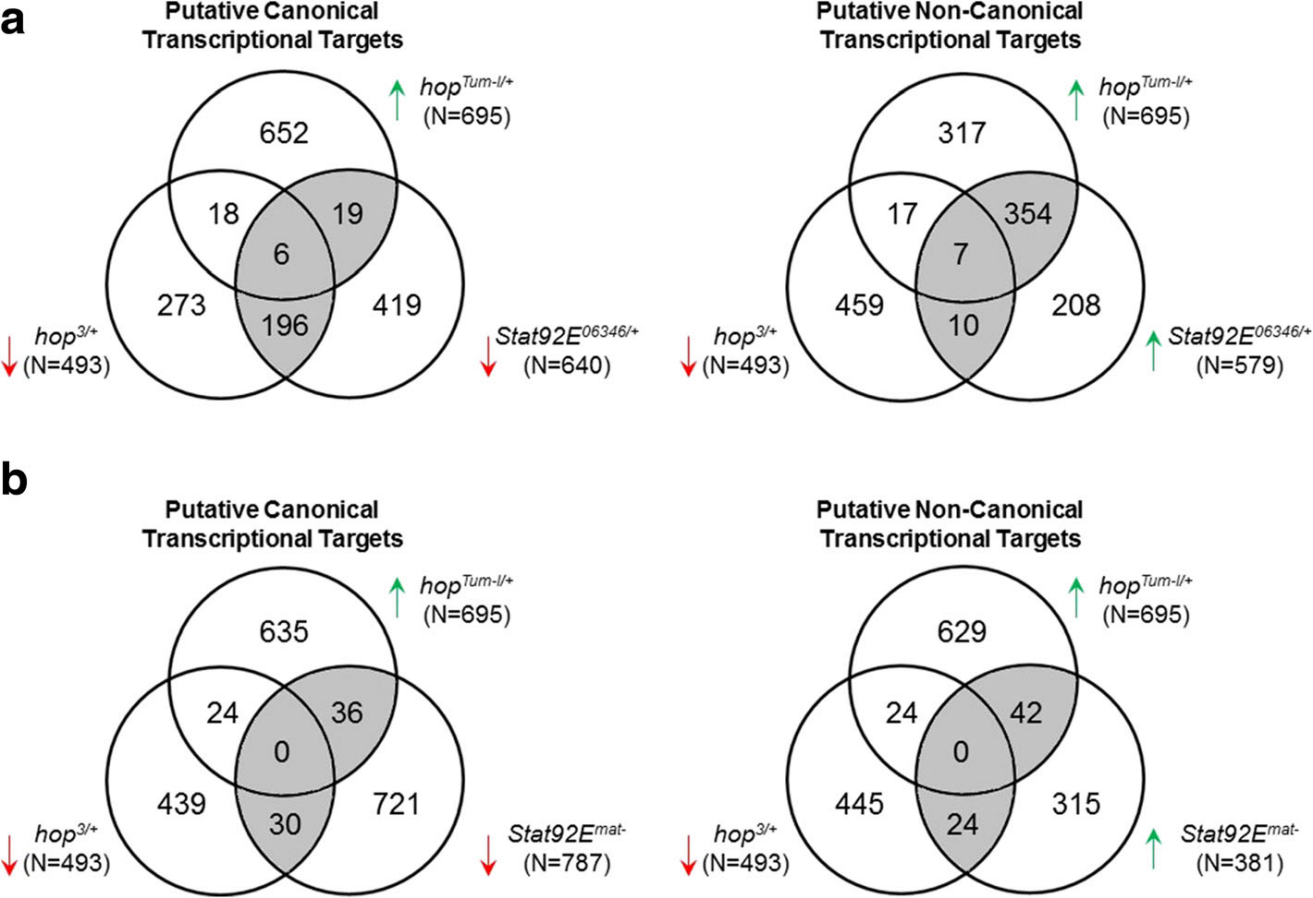
Biologically relevant overlapping probe sets of putative canonical and non-canonical transcriptional targets inferred by microarray. Significant genes were assessed for overlaps among the *Jak-Stat* mutants previously determined to be relevant for the canonical versus non-canonical heterochromatin-mediated silencing mechanism [19, 26] for (**a**) embryo samples and (**b**) maternal null and embryo samples

Since the hierarchical clustering analysis showed large differences in overall transcriptomes of *Stat92E* ^*mat-*^ and *Stat92E* ^*06346/+*^ embryos, we aimed to differentiate between canonical versus non-canonical JAK/STAT pathway by analyzing significantly regulated probe sets of *Jak* gain- (*hop* ^*Tum-l/+*^) and loss-of-function (*hop* ^*3/+*^) and *Stat* loss-of-function *(Stat92E* ^*06346/+*^) separately from the maternal null (*Stat92E* ^*mat-*^). For the putative canonical targets assessing overlaps among *hop* ^*Tum-l/+*^ upregulated, *hop* ^*3/+*^ downregulated and *Stat92E* ^*06346/+*^ downregulated genes, we found 221 putative probe sets and for the putative non-canonical targets assessing overlaps among *hop* ^*Tum−/+l*^ upregulated, *hop* ^*3/+*^ downregulated and *Stat92E* ^*06346/+*^ upregulated genes, we found 371 putative probe sets (Fig. 2A). While comparing putative canonical targets by assessing overlaps among *hop* ^*Tum-l/+*^ upregulated, *hop* ^*3/+*^ downregulated and *Stat92E* ^*mat-*^ downregulated genes, we found 66 putative probe sets and for the putative non-canonical targets assessing overlaps among *hop* ^*Tum-l/+*^ upregulated, *hop* ^*3/+*^ downregulated and *Stat92E* ^*mat-*^ upregulated genes, we found another 66 putative probe sets (Fig. 2B). The total number of unique probe sets for further analyses was 258 putative canonical and 409 putative non-canonical probe sets (Additional file 5: Table S4).

### Non-canonical targets are significantly more enriched with heterochromatin markers

**Fig. 3:**
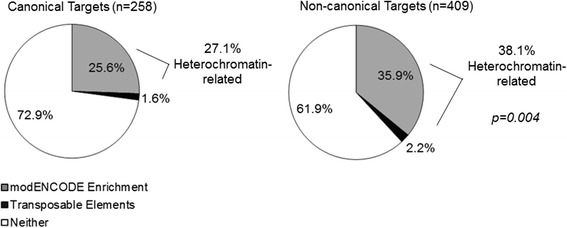
Non-canonical target sites have significantly more overlaps with heterochromatin sites. For both embryonic and maternal transcripts, overlaps between genomic loci corresponding to the probe set annotation and HP1a, Su(var)3–9 and/or H3K9me3 enriched loci listed in the relevant modENCODE database were tabulated. Probe sets annotated as transposable elements were also considered as heterochromatin sites. Non-canonical target probe sets had significantly higher proportion of such heterochromatin-related sites compared to canonical targets (*p* = 0.004, Fisher’s exact two-tailed test)

Since the distinct mode of the non-canonical JAK/STAT pathway involves heterochromatin stabilization by unphosphorylated STAT by its interaction with HP1a, we hypothesized that we would observe increased association of putative non-canonical target loci with key heterochromatin markers, HP1a, Su(var)3–9 and H3K9me3 [19, 26, 27, 29]. We therefore identified overlaps with the publicly available modENCODE ChIP-seq database annotating loci enriched with HP1a, Su(var)3–9 and H3K9me3 in various wildtype background samples at different developmental stages and *Drosophila* cell culture [56]. Among probe sets that were classified as putative canonical targets by our transcriptional analyses, 25.6% had HP1a, Su(var)3–9 and/or H3K9me3 enriched site overlap and 1.6% mapped to transposable elements, compared to 35.9% and 2.2% respectively in non-canonical targets. Our results comparing the putative canonical versus the putative non-canonical targets we identified from our transcriptome analyses thus indicate that as expected, there was significant difference between these two groups, in which the putative non-canonical transcriptional target group had a larger number of overlap sites with HP1a, Su(var)3–9 and/or H3K9me3, or are transposable element sites, compared to the group classified as canonical transcriptional targets (38.1% versus 27.1%, *p* = 0.004 Fisher’s exact test) (Fig. 3).

### Canonical and non-canonical targets have distinct biological roles

**Fig. 4:**
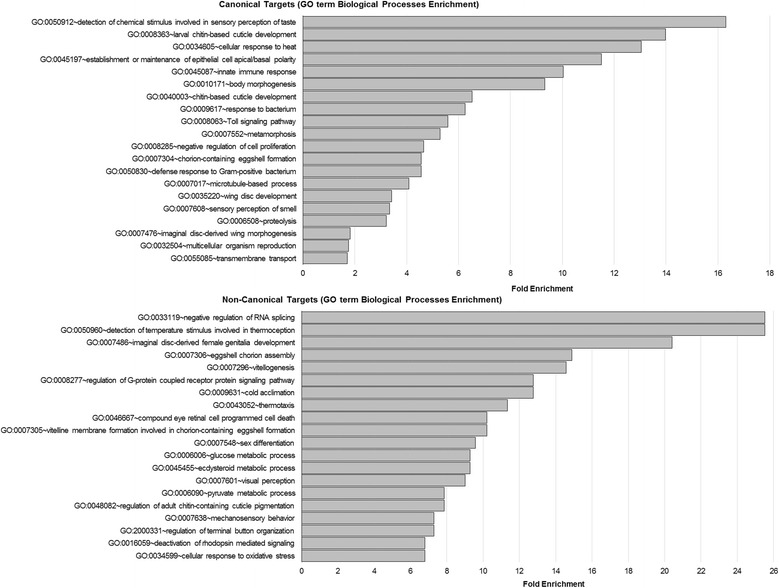
Canonical transcriptional targets are generally enriched with biological processes relevant to development and innate immunity, whereas non-canonical transcriptional targets appear to be involved with metabolism. (**a**) Gene Ontology (GO) enrichment of Biological Processes terms was assed using DAVID and the 20 highest fold enrichment GO terms and (**b**) all hits of KEGG pathway enrichment terms are shown for the canonical versus non-canonical target probe sets, in order of fold enrichment

After having classified probe sets to canonical versus non-canonical JAK/STAT pathway transcriptional target genes, we sought the biological significance of these different targets. We used the DAVID Gene Ontology database to determine biological functions and pathways that are enriched for the canonical and non-canonical target gene panel [57, 58]. Canonical targets appeared to be mainly involved in development and innate immunity, as expected for their well-established role, as seen by the top 20 fold enrichment of GO Biological Functions terms (Fig. 4). Ten out of 20 top enriched terms were relevant to development, including “larval chitin-based cuticle development,” “establishment of epithelial cell apical/basal polarity,” “body morphogenesis,” “chitin-based cuticle development,” “metamorphosis,” “negative regulation of cell proliferation,” “chorion-containing eggshell formation,” “wing disc development,” “imaginal disc-derived wing morphogenesis” and “multicellular organism reproduction.” Terms relevant to innate immune response were also found four times, including “innate immune response,” “response to bacterium,” “Toll signaling pathway” and “defense response to Gram-positive bacterium.” Other enrichment terms suggested a role for the canonical pathway in olfactory and sensory response, including “detection of chemical stimulus involved in sensory perception of taste,” and “sensory perception of smell.” Additional GO terms suggested transport mechanisms, including, “microtubule-based process” and “transmembrane transport” and others included “cellular response to heat” and “proteolysis.”

On the other hand, non-canonical targets were enriched mainly with terms relevant to metabolism, including “glucose metabolic process,” “ecdysteroid metabolic process,” “pyruvate metabolic process,” and stress response including “detection of temperature stimulus involved in thermoception,” “cold acclimation,” “thermotaxis,” “mechanosensory behavior,” and “cellular response to oxidative stress.” Female sex and egg development also appeared to be prevalent among the GO enriched terms, including “imaginal disc-derived female genitalia development,” “eggshell chorion assembly,” “vitellogenesis” and “vitelline membrane formation involved in chorion-containing eggshell formation.” Regulation of visual perception also appeared multiple times including “compound eye retinal cell programmed cell death,” “visual perception” and “deactivation of rhodopsin mediated signaling.” “Regulation of G-protein coupled receptor protein signaling pathway” was also among the top fold enrichment. The observation that the highest GO term fold enrichment was seen for “negative regulation of RNA splicing” is noteworthy, and may be related to the role of the non-canonical pathway in epigenetic signaling. GO terms related to chorion formation were found in both canonical and non-canonical targets, suggesting that while a large number of target loci distinct to canonical or non-canonical pathways exist, there is also a possibility of shared processes and targets.

## Discussion

Our current study aimed to differentiate between canonical versus non-canonical JAK/STAT target genes in *Drosophila* by using a genome-wide transcriptional analysis approach. The use of the *Drosophila* system with a simple JAK/STAT system that involves only one JAK and one STAT with mutant lines readily available facilitated this current study that aimed to distinguish between the two pathways by transcriptional analysis. The genetic analysis of the non-canonical JAK/STAT pathway and the establishment of the paradigm of unphosphorylated STAT as a key player in epigenetic regulation in the nucleus has been conducted in *Drosophila* [19, 26]. We therefore performed genome-wide transcriptome analyses to characterize transcriptional targets of relevant *Jak* and *Stat* mutants in this study. Furthermore, we quantified overlaps with ChIP-seq peaks of the major heterochromatin factors of HP1a, Su(var)3–9 and H3K9me3 binding sites using available modENCODE data. Our findings that putative non-canonical transcriptional targets had significantly more heterochromatin marker overlaps compared to the putative canonical targets given this study design supports the notion that while canonical targets rely mainly on classic signaling transduction modes to activate transcriptional target genes via phosphorylated and dimerized STAT, the non-canonical epigenetic mechanism via unphosphorylated STAT operates in a distinct manner, by its interaction with HP1a and heterochromatin associated factors. Additional studies conducting chromatin immunoprecipitation experiments in conjunction with transcriptomic analyses using samples of the exact same developmental stage would confirm the conclusions drawn from this study.

In flies and other organisms, HP1a-mediated heterochromatin maintenance has been implicated as a regulator of the aging process [28, 51]. A specific underlying association between heterochromatin regulation, metabolism, aging and oncogenesis can be inferred from multiple studies in different organisms. In *S. cerevisiae*, metabolism, genomic instability and cell life span through replication have been linked to heterochromatin [59]. Furthermore, it was found from a later study in *C.elegans*, that mutants of the HP1a homologue, HPL-2 altered germline gene expression that controls the switch to the dauer state, as well as longevity and lipid metabolism [60]. These observations are consistent with the GO term enrichment of the non-canonical JAK/STAT pathway we observed in the current study, in which we found among the highest fold enrichment, gene groups related to various metabolic pathways, stress response, the female genitalia and egg development. It has been shown previously that unphosphorylated STAT can be protective against genome instability and oncogenesis by its interaction with HP1a, and may further suggest that the enrichment in various metabolism-related genes we have observed especially in the non-canonical heterochromatin-mediated pathway may be related to the aforementioned links among these various processes described in different species [27, 29]. Stress response terms were also frequently found to be among the top 20 fold enrichment, mostly regarding cold temperature sensing and response, as well as response to oxidative stress. It has been shown previously in *Arabidopsis* that epigenetic regulation of heterochromatin markers occur at repetitive elements in response to cold-stress, as well as activation of transcription of heterochromatic loci induced by stress [61, 62]. Furthermore, a link between oxidative stress and heterochromatin stabilization has been described and shown to provide genome protection [63]. It is also interesting to note that the highest fold enrichment GO term found among non-canonical targets was “negative regulation of RNA splicing.” Considering the epigenetic role of the non-canonical pathway, it is conceivable that additionally to heterochromatin maintenance, the non-canonical pathway may coordinate RNA splicing. In contrast to the putative non-canonical targets where GO terms related to metabolism, stress response and female sex-related terms were among the most enriched terms, GO term enrichment of the canonical target gene group were related to the well-established roles of the JAK/STAT pathway, development and innate immune response, as expected. Transcriptional regulation of genes related to development and innate immunity by the JAK/STAT pathway is known to be highly conserved across species [12, 64]. Indeed, we found *TotC* and *TotX* in our analyses of putative transcriptional target genes inferred from the microarray and modEncode overlaps (Additional file 5: Table S4). These observations provide further support for the idea that our approach may be effective in uncovering additional target genes, both canonical and non-canonical.

In this study, we utilized relatively young mutant embryos laid between 0 and 12 h. On the other hand, the modENCODE dataset that we inferred heterochromatin-related factors varied, from early embryo to the late larval stage of different genetic backgrounds, as well as various cell lines. Our conclusions would therefore be strengthened by additional experimental studies using specific genetic backgrounds, same tissues and temporally regulated samples for further transcriptional analyses, coupled by chromatin immunoprecipitation of relevant heterochromatin factors of the corresponding samples. Such future studies would enable us to identify additional targets, and perform an increasingly meticulous analysis to differentiate between the canonical versus non-canonical JAK/STAT pathway. Nevertheless, our current study supports the notion that canonical versus non-canonical JAK/STAT pathway may regulate numerous biologically distinct processes additionally to some possible overlaps and it would be beneficial to conduct further studies that establish methods that distinguish between the two pathways.

## Conclusions

We conducted a genome-wide analysis comparing putative canonical versus non-canonical JAK/STAT pathway transcriptional targets during early *Drosophila* development. Our findings of differences between canonical versus non-canonical JAK/STAT pathway targets and specific loci regulated across the genome give us insights into the significant and distinct biological roles of each that may exist, additionally to possible overlapping targets.

## Methods

### Fly stocks/genetics and RNA sample preparation

All crosses were carried out at 25 °C on standard cornmeal/agar medium. Fly stocks of *w* ^*1118*^, *hop*3*/FM7c*, *Stat92E* ^*06346*^ */TM3, FRT* ^*82B*^ *[ovo* ^*D1*^ *, w* ^*+*^ *]/TM3,* and *hop* ^*Tum-l*^ */FM7c* were obtained from the Bloomington *Drosophila* Stock Center (Bloomington, IN). For the preparation of heterozygous embryos, females from the mutant stocks were crossed with *w* ^*1118*^ males and the resulting *hop* ^*3/+*^, *Stat92E* ^*06346/+*^ or *hop* ^*Tum-l/+*^ progeny were used to produce embryos, which were collected between 0 and 12 h after egg laying on apple agar plates with yeast paste. To generate *Stat92E* ^*mat–*^ embryos, *hsp70-flp; FRT* ^*82B*^ *Stat92E* ^*06346*^ */TM3* females were crossed to *hsp70-Flp; FRT* ^*82B*^ *[ovo* ^*D1*^ *, w* ^*+*^ *]/TM3* males. Third instar larval progenies were heat-shocked at 37 °C for 2 h daily over 3 to 4 days, and resulting adult females of the genotype *hsp70-flp; FRT* ^*82B*^ *Stat92E* ^*06346*^ */FRT* ^*82B*^ *[ovo* ^*D1*^ *, w* ^*+*^ *]* which were used to produce embryos lacking in maternal *Stat92E* gene products, as described previously in the dominant female-sterile “germline clone” technique [65]. *Stat92E* ^*mat–*^ and control *w* ^*1118*^ were collected between 1 and 2 h after egg laying on apple agar plates with yeast paste. The embryos were washed twice with deionized water and total RNA was prepared using the RNeasy Plus Mini kit (Qiagen) according to the manufacturer’s manual. RNA quality was assessed using the Agilent 2100 Bioanalyzer and the RNA 6000 Nano kit (Agilent Technologies Inc., Palo Alto, CA).

### Microarray analyses

To prepare microarray samples from the RNA prepared, 200 ng of total RNA was used to prepare biotin-labeled RNA using Ambion MessageAmp Premier RNA Amplification Kit (Applied Biosystems, Forster City, CA). Briefly, the first strand of cDNA was synthesized using ArrayScript reverse transcriptase and an oligo(dT) primer bearing a T7 promoter. Then DNA polymerase I was used (in the presence of *E. coli* RNase H and DNA ligase) to convert single-stranded cDNA into double-stranded DNA (dsDNA), which was then used as a template for in vitro transcription in a reaction containing biotin-labeled UTP and T7 RNA Polymerase to generate biotin-labeled antisense RNA (aRNA). Twenty μg of labeled aRNA was fragmented and 15 μg of the fragmented aRNA was hybridized to Affymetrix *Drosophila* Genome 2.0 Array Chips according to the manufacterer’s Manual (Affymetrix, Santa Clara, CA). Array Chips were stained with streptavidin-phycoerythrin, followed by an antibody solution (anti-streptavidin) and a second streptavidin-phycoerythrin solution, performed by a GeneChip Fluidics Station 450. The Array Chips were scanned with the Affymetrix GeneChip Scanner 3000. For the numerical conversion to expression intensity and Present/Absent calls employing MAS5 [66] (Additional file 1: Table S1), the Genespring software (Agilent Technologies Inc., Palo Alto, CA) or the R package Affy was used [67, 68]. R version 3.1.3 was used for the analyses.

For each mutant genotype, control probe sets were filtered, as well as those where the wild-type and respective mutant intensities all had the “Absent” call. The top 10th percentile upregulated and downregulated *log*_*2*_ fold change of all probes were found to be 1.027 and −1.047, respectively and therefore the 2-fold change cut-off was considered to be significantly differentially regulated genes for each mutant genotype (Additional file 2: Table S2). Pearson’s correlation with complete distance separation was used for the clustering and heatmap representation of the differentially regulated probe sets. For conducting RT-PCR, 0–12 h embryos were collected and total RNA was harvarested using the RNeasy kit (Qiagen). The SuperScript™ III Reverse Transcriptase kit (Invitrogen) was used to generate cDNA as a template for semi-quantitative PCR.

HP1a and Su(var)3–9 binding and the heterochromatic H3K9me3 enriched sites were obtained from the publicly available modENCODE ChIP-seq database for comparison with genomic sites associated with the relevant probe sets identified from the microarray analysis [56]. The following modENCODE data files were used: for HP1a binding sites, #3956 (Oregon^R^ 14–16 h embryo), #323 (S2 cells), #955 (Oregon^R^ 3rd instar larvae), #2074 (S2 cells), #2665 (Oregon^R^ 2–4 h embryo), #2666 (BG3-c2 cells), #2668 (S2 cells) and #3956 (Oregon^R^ 14–16 h embryo), for Su(var)3–9 binding sites, #952 (BG3-c2) and #2673 (S2), and for H3K9me3 enrichment, #971 (yellow cinnabar brown speck 0–4 h embryo) and #4939 (Oregon^R^ 14–16 h embryo). In our study, if one or more enrichment sites were found to overlap with the annotated genomic locus indicated by the microarray data, the gene was deemed to be relevant to heterochromatin. For the overlap between probe sets upregulated by *hop* ^*Tum-l/+*^ and downregulated by *hop* ^*3/+*^, probe sets overlapping with downregulated *Stat92E* ^*06346/+*^ was categorized as putative canonical targets, whereas those upregulated were categorized as non-canonical targets. Similarly, *Stat92E* ^*mat-*^ was also analyzed by taking into consideration, overlaps with *hop* ^*Tum-l/+*^ upregulated and *hop* ^*3/+*^ downregulated probe sets. Probe sets where the *Stat92E* ^*06346/+*^ and *Stat92E* ^*mat-*^ showed opposite trends were removed from the analyses, as well as *hop* ^*Tum-l/+*^ and *hop* ^*3/+*^ overlaps without significant *Stat92E* ^*06346/+*^ or *Stat92E* ^*mat-*^ differential expression.

The two-tailed Fisher’s exact test was used to determine significance between the difference in the number of probe sets for which their relevant sites overlapped with HP1a, Su(var)3–9 and/or H3K9me3 enriched sites and transposable elements comparing putative canonical versus non-canonical targets. Database for Annotation, Visualization and Integrated Discovery (DAVID), version 6.8 Beta was used for functional annotation and assessing the top 20 Fold Enrichment of Gene Ontology terms of putative canonical and non-canonical sets [57].

## Additional files

Additional file 1: Normalized dataset. (XLSX 3589 kb)

Additional file 2: Significant up- and down-regulated fold change probe sets of each mutant genotype. (XLSX 308 kb)

Additional file 3: Overlaps of significantly changed mutant probe sets and putative canonical/non-canonical transcriptional target classification. (XLSX 138 kb)

Additional file 4: Fig. S1. Validation of transcriptional upregulation of known target genes in *hop* ^*Tum/+*^ embryo samples. RT-PCR was conducted on 0–12 h *W* ^*1118*^ wildtype control, or *hop* ^*Tum/+*^ embryo collection to assess the upregulation of previously known JAK-STAT target genes. (JPEG 27 kb)

Additional file 5: Heterochromatin marker modENCODE enrichment and transcriptional target overlaps. (XLSX 56 kb)

## Abbreviations

ChIP-seq: Chromatin immunoprecipitation-sequencing
H3K9me3: Histone 3 Lysine 9 tri-methylation
HP1a: Heterochromatin Protein 1a
JAK/STAT: Janus kinase-signal transducer and activator of transcription
Su(var)205: Suppressor of varigation 205
