# Supplementary figures and images for: Canonical and non-canonical JAK/STAT transcriptional targets may be involved in distinct and overlapping cellular processes
Source: BMC Genomics. 2017 Sep 11;18:718. doi: 10.1186/s12864-017-4058-y (PMC5594485; doi:10.1186/s12864-017-4058-y)

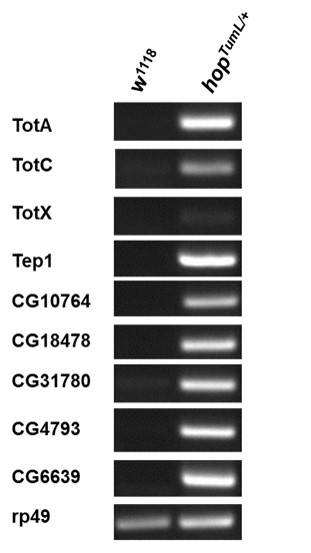

Supplement: Supplementary file 4 — Validation of transcriptional upregulation of known target genes in hop Tum/+ embryo samples. RT-PCR was conducted on 0–12 h W 1118 wildtype control, or hop Tum/+ embryo collection to assess the upregulation of previously known JAK-STAT target genes. (JPEG 27 kb) [file 12864_2017_4058_MOESM4_ESM.jpg]
